# Active Compensation of Radiation Effects on Optical Fibers for Sensing Applications

**DOI:** 10.3390/s21248193

**Published:** 2021-12-08

**Authors:** Sohel Rana, Austin Fleming, Nirmala Kandadai, Harish Subbaraman

**Affiliations:** 1 Measurement Science Department, Idaho National Laboratory, 1955 N Fremont Avenue, Idaho Falls, ID 83415, USA; sohel.rana@inl.gov; 2 Department of Electrical and Computer Engineering, Boise State University, Boise, ID 83725, USA; nirmalakandadai@boisestate.edu (N.K.); harishsubbaraman@boisestate.edu (H.S.)

**Keywords:** active compensation, radiation-induced attenuation, radiation-induced compaction, cascaded Fabry–Perot interferometer

## Abstract

Neutron and gamma irradiation is known to compact silica, resulting in macroscopic changes in refractive index (RI) and geometric structure. The change in RI and linear compaction in a radiation environment is caused by three well-known mechanisms: (i) radiation-induced attenuation (RIA), (ii) radiation-induced compaction (RIC), and (iii) radiation-induced emission (RIE). These macroscopic changes induce errors in monitoring physical parameters such as temperature, pressure, and strain in optical fiber-based sensors, which limit their application in radiation environments. We present a cascaded Fabry–Perot interferometer (FPI) technique to measure macroscopic properties, such as radiation-induced change in RI and length compaction in real time to actively account for sensor drift. The proposed cascaded FPI consists of two cavities: the first cavity is an air cavity, and the second is a silica cavity. The length compaction from the air cavity is used to deduce the RI change within the silica cavity. We utilize fast Fourier transform (FFT) algorithm and two bandpass filters for the signal extraction of each cavity. Inclusion of such a simple cascaded FPI structure will enable accurate determination of physical parameters under the test.

## 1. Introduction

Radiation exposure of vitreous silica can induce changes in density up to 3% [1,2,3], whereas pressure can alter it more than 20% [4,5,6]. However, it has been shown that depending on the amount of pressure applied in the presence of temperature, the change in density in the silica glass can either be temporary or permanent [7,8]. On the other hand, radiation-induced changes in density are always irreversible [1,9] and follows a power law for dose-dependent exposure given by [3]:

(1) $$\frac{\Delta \rho }{\rho }=AD^{c}$$

where ρ and D are the density and absorbed radiation dose, respectively and A, and c are material-dependent constants. The value of constant c has been found $c≅\frac{2}{3}$ for the ionization process (gamma ray, X-ray, UV ray, etc.) [1,10,11,12,13,14,15,16] and c≅1 for atomic displacement process (neutron, He^+^, D^+^, etc.) [1]. While there might be many reasons for different values of c, it has been shown that it is the Si-O-Si bond angles rather than the change in the shape of silica tetrahedron (SiO_4_) undergoes significant changes due to particle bombardment or under high pressure [17,18]. To validate the different values of c in Equation (1), Piao et al. [3] reported a two phase structural model for vitreous silica. It has been found that neutron irradiation causes a significant compaction compared to gamma irradiation [1,11]. The detailed discussion about the mechanism of radiation-induced compaction in silica can be found in Ref [19]. It has been reported that during the fast neutron fluence in excess of $\sim 10^{19}\;n/cm^{2}$, silica reaches the metamict phase with an increased density change between 2–3% [1,20]. Further fluence of neutrons does not further increase the density.

Radiation, primarily through radiation-induced attenuation (RIA) and radiation-induced compaction (RIC) within silica optical fibers, changes the optical, mechanical, and chemical properties of silica fiber in many ways, thus affecting signal fidelity. Specifically, these radiation-induced effects in silica induces an error in predominantly used resonance based optical fiber sensors (OFS), such as fiber Bragg grating (FBG), long period grating (LPG), and Fabry–Perot (F-P) in measuring physical parameters like temperature, pressure, strain, etc. For example, recent works on FBGs [21,22], LPGs [23,24], and F-Ps [25,26] sensors in a gamma and mixed radiation (gamma and neutron radiation) environment explained radiation-induced drift in detail.

RIA increases the linear attenuation in silica based fibers [27,28]. Different parameters govern the RIA levels and kinetics, which include chemical compositions of fibers [29,30], manufacturing process [31,32,33], light guiding properties of fibers, the nature of irradiation (X-ray, gamma ray, neutron, etc.) [34], the dose rate [34,35,36], wavelength of light used [37,38], injected light power [39], temperature of irradiation [40,41], etc. On the other hand, RIC causes the structural changes of the fiber, leading to a density change [1]. While RIA leads to RI change through the Kramer–Kronig relation [42,43], its determination is complex and one needs to consider the spectrum over a wide frequency range [43]. RIC alters the RI through Lorentz–Lorenz [44,45] and point dipole theory [46]. So far, radiation-induced compaction has been calculated using power law [3] and empirical equation [1]. Then, Lorentz–Lorenz relationship, point dipole theory, sensitivity factor, and a few empirical equations have been utilized to find out the change in RI and linear compaction from the volume compaction [3,9,44,46,47,48]. An immediate question arises on whether these methods (Lorentz–Lorenz, point dipole theory) consider the combined RIC, RIA and dopant diffusion effects on the RI change or only consider the RIC effects on the RI change. In this regard, these methods do not present the whole picture regarding RI change due to radiation. As RI and length compaction are the input parameters for the resonance-based OFS, accurate calculation of these parameters is of great importance to predict the actual radiation effects on OFS and correct the sensor drift.

An in-line measurement of RI and length changes due to radiation can be a potential way to understanding the structural change of optical fiber in a nuclear environment, thus helping in minimizing signal error. Such in-line measurement techniques must provide the change in RI of optical fiber due to any specific phenomena the fiber is subjected to, including RIC, RIA, dopant diffusion, temperatures, dose, and dose rate [9,21,22,41]. Once the macroscopic properties are measured, it is comparatively easy to understand how they would impact the sensor performance; therefore, enabling real-time correction of sensor signal drifts.

**Figure 1:**
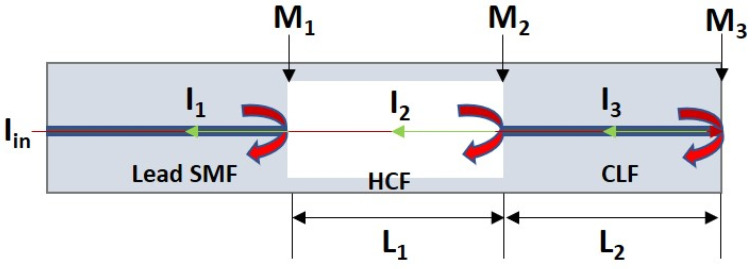
Schematic of the designed cascaded Fabry–Perot interferometer.

In this paper, we propose a simple cascaded Fabry–Perot sensor to measure the radiation-induced change in RI along with length compaction due to radiation for active compensation of signal drift. Compared to conventional cook and look method, analytical method using Lorentz–Lorenz, point dipole theory, etc., this technique provides unique features, such as real-time determination of RI and length changes due to any specific phenomena the fiber is subjected to, including RIC, RIA, dopant diffusion, temperatures, etc. Our proposed cascaded Fabry–Perot structure consists of a hollow cavity (air/gas cavity) and a solid cavity (silica cavity) within the same fiber, as shown in Figure 1. The air cavity can be used to measure the radiation-induced linear compaction from the spectral response since RI shows no or little change. However, radiation alters both the length and the RI in the silica cavity. Since the silica capillary tube and the silica cavity are both made of silica, and both cavities are in very close proximity to each other, it is expected that both the cavities will experience the same compaction. Considering the same amount of linear compaction in both cavities, change in RI can be measured from the spectral response of the silica cavity. Separation of the signal for each cavity is required to calculate the change in RI of the silica cavity with the help of the air cavity. We use fast Fourier transform (FFT) algorithm to convert the wavelength domain signal into frequency domain. Then, two bandpass filters are applied to separate the signals of each cavity. Finally, inverse FFT is used to convert the filtered signals into wavelength domain. This simple way of finding the radiation-induced change in RI and linear compaction simultaneously will help improve understanding of the radiation effects on OFS.

## 2. Geometric Structure and Principles

**Figure 2:**
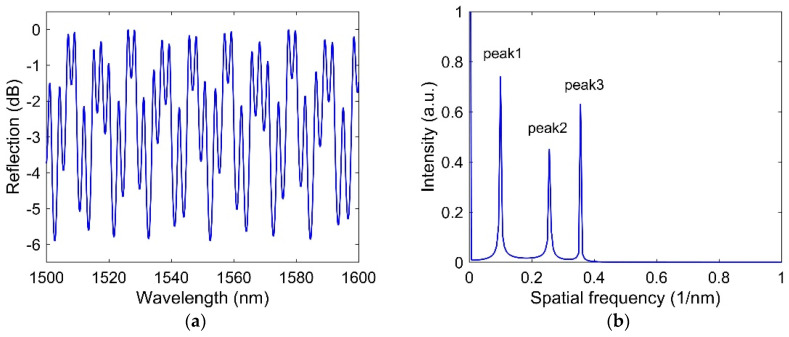
(**a**) total spectrum of the cascaded FPI and (**b**) the spatial frequency distribution of the total spectrum using FFT.

The cascaded FPI, shown in Figure 1, consists of a hollow silica capillary fiber (HCF) of length $L_{1}$ spliced between a coreless silica fiber (CLF) of length $L_{2}$ and a lead single mode fiber (SMF) of arbitrary length. The air cavity has a cavity length ($L_{1}$) of 117 µm and refractive index ($n_{1}$) of 1. The silica cavity has a cavity length ($L_{2}$) of 211 µm and refractive index ($n_{2}$) of 1.44402. The value of refractive $n_{2}$ has been calculated by using the three-term Sellmeier Equation [49] at 1550 nm of wavelength. The cavity lengths were chosen in such a way so that the signal peak of each cavity does not overlap each other in spatial frequency domain. There is one more cavity that consists of $L_{1}+L_{2}$ and is sometimes referred to as a hybrid cavity (air-silica cavity). The main components of this cascaded FPI are three reflective interfaces, $M_{1}$ (between the interface of lead SMF and HCF), $M_{2}$ (between the interface of end facet HCF and CLF), and $M_{3}$ (between the interface of end facet CLF and air). The total interference spectrum from the cascaded FPI is a three-beam interference and can be expressed by [50]

(2) $$I=I_{1}+I_{2}+I_{3}+2\sqrt{I_{1}I_{2}}\cos\left(\varphi_{air}\right)+2\sqrt{I_{2}I_{3}}\cos\left(\varphi_{silica}\right)\;+2\sqrt{I_{1}I_{3}}\cos\left(\varphi_{air-silica}\right)$$

where $\varphi_{air}=\frac{4\pi n_{1}L_{1}}{\lambda }$, $\varphi_{silica}=\frac{4\pi n_{2}L_{2}}{\lambda }$, and $\varphi_{air-silica}=\frac{4\pi }{\lambda }\left(n_{1}L_{1}+n_{2}L_{2}\right)$ are the phase of the air cavity (first cavity), silica cavity (second cavity), and hybrid cavity, respectively. The total spectrum from the cascaded FPI is shown in Figure 2a. To separate the reflection spectrum of each cavity, optical frequency domain signal processing was used to retrieve the air cavity and the silica cavity. The fast Fourier transform (FFT) algorithm is applied to the total spectrum, and the corresponding spatial frequency domain distribution is shown in Figure 2b. It is seen that three peaks are available in the frequency domain. These three peaks are situated at 0.1 ${\mathrm{nm}}^{-1}$ (peak 1), 0.2562 ${\mathrm{nm}}^{-1}$ (peak 2), and 0.3562 ${\mathrm{nm}}^{-1}$ (peak 3). The spatial frequency values of the air cavity, silica cavity, and hybrid cavity are calculated as $f_{1}=\frac{2n_{1}L_{1}}{\lambda_{1}\lambda_{2}}$, $f_{2}=\frac{2n_{2}L_{2}}{\lambda_{1}\lambda_{2}}$, and $f_{1}+f_{2}$, respectively, where $\lambda_{1}$ and $\lambda_{2}$ are the wavelengths of adjacent peaks or dips in the reflection spectrum of each cavity. Calculation of the spatial frequency for each cavity indicates that peak 1, peak 2, and peak 3 correspond to the peaks of air cavity, silica cavity, and hybrid cavity, respectively.

**Figure 3:**
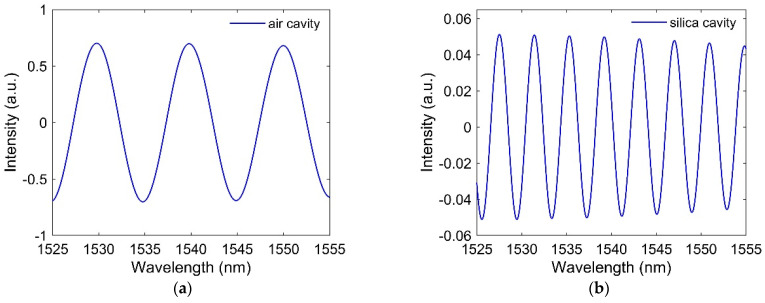
Retrieved signals using bandpass filters and inverse FFT for (**a**) air cavity and (**b**) silica cavity.

The spatial frequencies of the air cavity ($f_{1}$) and the silica cavity ($f_{2}$) are related to the optical path difference (OPD) of these two cavities. The crosstalk between the two cascaded cavities can be reduced by increasing the OPD between the air cavity and the silica cavity. To extract the signals for each cavity from the total reflection spectrum, two bandpass filters are used. The interference spectrum of the air cavity is obtained by filtering the total spectrum using a bandpass filter centered on peak1 and for the silica cavity centered on peak 2. Then, the lengths of the air cavity and silica cavity can be obtained by using $L_{1}=\frac{\lambda_{1}\lambda_{2}}{2n_{1}\left(\lambda_{1}-\lambda_{2}\right)}$ and $L_{2}=\frac{\lambda_{1}\lambda_{2}}{2n_{2}(\lambda_{2}-\lambda_{1})}$, respectively, if $n_{1}$ and $n_{2}$ are known. The reconstructed spectrum for the air cavity and silica cavity is shown in Figure 3a,b, respectively. Table 1 shows the original and reconstructed length of the cavities. It is seen that the retrieved lengths of the cavities are almost same as that of the original length of the cavities. There is one more term called free spectral range (FSR), which is the spectral distance between two adjacent peaks or dips. This is calculated for the air cavity and the silica cavity as $FSR_{air}=\frac{\lambda_{1}\lambda_{2}}{2n_{1}L_{1}}$ and $FSR_{silica}=\frac{\lambda_{1}\lambda_{2}}{2n_{2}L_{2}}$.

**Figure 4:**
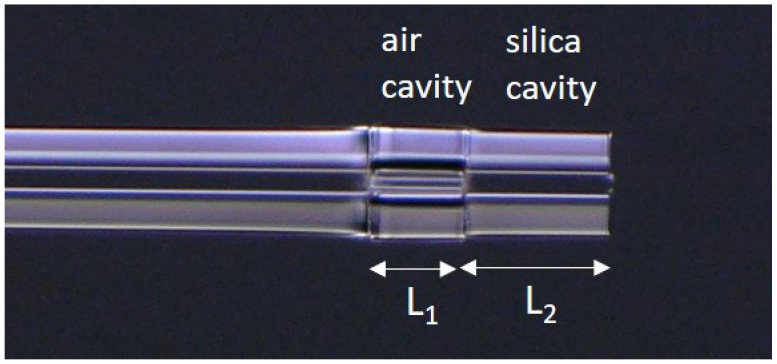
Microscopic image of the fabricated cascaded FPI.

Although this paper focuses on theoretical and numerical analyses, we fabricated a cascaded FPI for better understanding. Since the cascaded FPI consists of SMF, HCF, and CLF, only cleaving and splicing are required to give the practical realization of this structure. We used an SMF-28 single-mode fiber as the lead-in fiber and capillary tubes with inner diameters of 39.2 µm (TSP040150) from Polymicro Technologies, in order to construct the air cavity. As a first step, we cleaved the SMF, HCF, and CLF using a cleaving tool (CT101/102, Fujikura) and then fusion spliced the SMF-28 with the HCF using a fusion splicer (70S + fusion splicer, Fujikura). Next, a linear stage in conjunction with the cleaving tool was used to cleave the spliced HCF at a certain distance from the splicing point. A CLF fiber from Thorlabs was cleaved and spliced with the capillary tube to complete the silica cavity. The microscopic image of the fabricated cascaded FPI is shown in Figure 4.

## 3. Compaction Analysis

Radiation changes both the length and the RI of the silica-based fiber. The volume compaction as a function of neutron fluence induced by radiation can be calculated by [1]

(3) $$C_{v}\left(\varphi \right)=C_{v}{}_{\infty }\left(1-e^{-\frac{\varphi }{\varphi_{S}}}\right)$$

where $C_{v}\left(\varphi \right)$ is the amount of compaction dependent on neutron fluence φ, $C_{v}{}_{\infty }$ is the equilibrium compaction, and $\varphi_{S}$ is the fluence at which the fiber material gets saturated. The linear compaction ($C_{l}$) can then be calculated from the $C_{v}$, assuming isotropic changes by the following empirical equation:

(4) $$C_{l}=1-{\left(1-C_{v}\right)}^{\frac{1}{3}}$$

Once the $C_{v}$ is known, the final density due to radiation can be calculated by using the following empirical equation:

(5) $$\frac{\rho_{2}-\rho_{1}}{\rho_{2}}=\frac{v_{1}-v_{2}}{v_{1}}=C_{v}$$

where $\rho_{1}$,$\;v_{1}$, $\rho_{2}$, and $v_{2}$ are the initial density, initial volume, final density, and final volume of the material, respectively. Once the density is known, the change in RI due to radiation-induced compaction can be calculated by using point dipole theory or Lorentz–Lorenz equation. The extended point dipole theory can be expressed as [46]

(6) $$\frac{n^{2}-1}{4\pi +b\left(n^{2}-1\right)}=\frac{\alpha }{M}\rho$$

and the Lorentz–Lorenz relationship can be represented by the following expression [44,45]

(7) $$\frac{n^{2}-1}{n^{2}+2}=\frac{4\pi }{3}N\alpha =\frac{4\pi }{3}\left(\frac{\rho N_{A}}{M}\right)\alpha$$

where *n*, N, ρ, M, α, *b*, and $N_{A}$, are the refractive indices of silica glass, number density of silica glass, mass density of silica glass, molecular weight of silica glass, electronic polarizability of silica glass, electronic overlap between adjacent dipoles in the silica glass, and Avogadro number, respectively. Since the proposed cascaded FPI consists of an air cavity and a silica cavity, the radiation-induced change in RI for both cavities would be different. As the two cavities are very close to each other, we can reasonably assume the change in length compaction is identical for both cavities since they will experience the same environment. The length compaction information is obtained from the interference spectrum of the air cavity as no change occurs in RI for being hollow cavity. If $L_{1i}$ is the intial cavity length and $L_{1f}$ is the compacted length of the air cavity due to the exposure of radiation, the linear companion ($C_{l-air}$) is

(8) $$C_{l-air}=\frac{L_{1i}-L_{1f}}{L_{1i}}$$

As the second cavity is based on silica, both the change in RI and length will occur. If $L_{2i}$ is the intial cavity length and $L_{2f}$ is the compacted length of the silica cavity due to the exposure of radiation, the linear compation ($C_{l-silica}$) is:

(9) $$C_{l-silica}=\frac{L_{2i}-L_{2f}}{L_{2i}}=C_{l-air}$$

As mentioned, the length compaction of the silica cavity would be same as that of the air cavity ($C_{l-silica}=C_{l-air}$). Based on that consideration, the compacted length of the silica cavity $L_{2f}$ can be calculated as

(10) $$L_{2f}=L_{2i}-C_{l-air}L_{2i}$$

It is known that optical length equals the physical length multiplied by refractive index of the medium. So, the optical length for the silica cavity, $L_{op}=n_{2f}L_{2f}$ where $n_{2f}$ is the compacted RI due to radiation. The optical length ($L_{op}$) of the silica cavity can be found from the interference spectrum of the silica cavity. As we already know, $L_{op}$ and $L_{2f}$, then $n_{2f}$ can be calculated easily. In this approach, compaction and RI can be identified independently and in real-time.

## 4. Radiation Effects on Cavities

**Figure 5:**
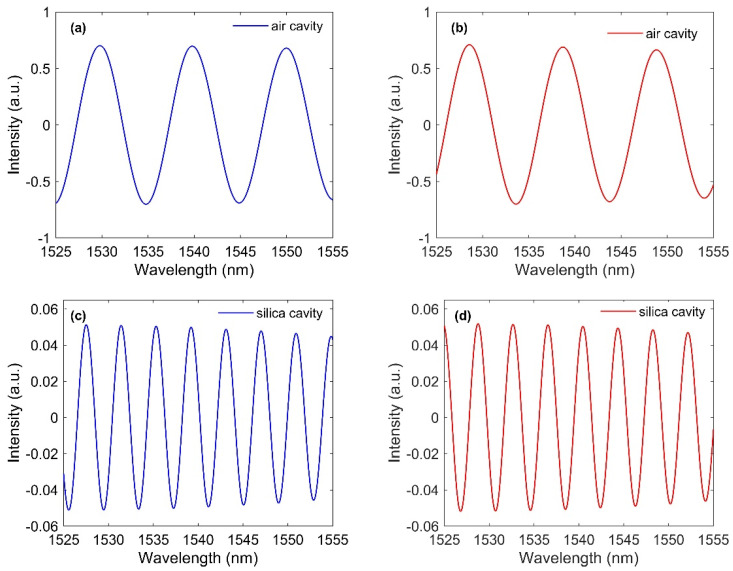
Reconstructed signals for (**a**) air cavity before irradiation, (**b**) air cavity after irradiation, (**c**) silica cavity before irradiation, (**d**) silica cavity after irradiation.

It is simple to calculate $C_{l-air}$ and $n_{2f}$ using Equations (8)–(10) from the measured fringe spectrum of a cascaded FPI. To test the validity of our proposed model, we used experimental values of linear and volumetric compaction reported in [9] where silica samples were exposed to high radiation field at different temperatures to observe the structural changes. The reported values for linear and volumetric compaction are 0.73 ± 0.04% and 2.20 + 0.13%, respectively, in Ref [9]. These values were measured after exposing the silica samples to a fast neutron fluence of $2.4\times 10^{21}\;n/{\mathrm{cm}}^{2}$ at a temperature of 95 °C. Point dipole theory (Equation (6)) is then used to calculate the final $n_{2f}$ from the density (volume compaction) based on the values reported in Ref [9]. We input the changes in RI and length due to a fast neutron fluence of $2.4\times 10^{21}\;n/{\mathrm{cm}}^{2}$ at a temperature of 95 °C to our cascaded FPI model. The individual cavity spectra from our simulations before and after irradiating to a fluence of $2.4\times 10^{21}\;n/{\mathrm{cm}}^{2}$ are shown in Figure 5. As the fiber is compacted, it is expected that the cavity lengths of both cavities will decrease in comparison to previous reconstructed cavity length. It is seen that there is a spectral change in Figure 5b,d due to the radiation effects from their original spectra Figure 5a,b. Table 2 shows the reconstructed length of cavities before and after the irradiation. It is seen that the reconstructed cavity lengths change from 117.39 to 116.56 µm for the air cavity and from 210.69 to 209.20 µm for the silica cavity. 

**Figure 6:**
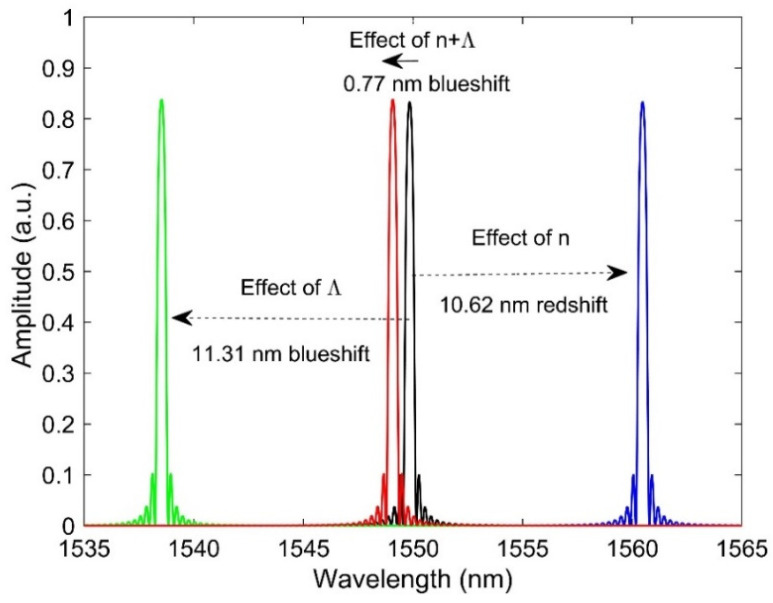
Spectra of FBG in different conditions: before irradiation (black curve), radiation-induced RI effect (blue curve), radiation-induced grating period effect (green curve), and radiation-induced combined effect of RI and grating period (red curve) on the Bragg wavelength.

Since the changes in RI and length due to radiation can be measured using cascaded FPI, it is possible to input these values into fiber Bragg grating (FBG) sensor to observe the radiation-induced signal drift. FBGs are periodic modulations of the refractive index of the core of a fiber with a typical period of less than 1 µm [51]. In an FBG, the fundamental guided mode couples to the counterpropagating guided mode when the following phase-matching condition is satisfied [52]:

(11) $$\lambda_{B}=2n_{eff}\Lambda$$

where $\lambda_{B}$ is the Bragg wavelength, $n_{eff}$ is the effective RI of the core, and Λ is the grating period. As the first step to comprehend the radiation effects on FBG, we modelled a FBG by using the theoretical analysis described in Ref [53]. The reflectivity of a uniform grating based on couple mode theory (CMT) can be expressed as

(12) $$R=\frac{sinh^{2}\left(\kappa L\sqrt{1-{\left(\frac{\delta }{\kappa }\right)}^{2}}\right)}{\left(1-{\left(\frac{\delta }{\kappa }\right)}^{2}\right)cosh^{2}\left(\kappa L\sqrt{1-{\left(\frac{\delta }{\kappa }\right)}^{2}}\right)+{\left(\frac{\delta }{\kappa }\right)}^{2}sinh^{2}\left(\kappa L\sqrt{1-{\left(\frac{\delta }{\kappa }\right)}^{2}}\right)}$$

where $\kappa =\frac{\pi }{\lambda_{Bragg}}\eta \Delta n^{mod}$ is the coupling coefficient, $\delta =\frac{2\pi n_{eff}}{\lambda }-\frac{\pi }{\Lambda }$ and $\frac{\delta }{\kappa }$ are the detuning ratio depending on $\frac{\lambda }{\lambda_{Bragg}}$, and $\Delta n^{mod}$ is the grating strength. The modal overlap factor (η), which defines the core power, can be expressed as [54]

(13) $$\eta =\frac{\pi^{2}\emptyset_{core}^{2}NA^{2}}{\lambda^{2}+\pi^{2}\emptyset_{core}^{2}NA^{2}}$$

where $\emptyset_{core}$ and NA are the core diameter and the numerical aperture of the fiber, respectively. We choose the parameters of the FBG in such a way to get the Bragg peak close to 1550 nm of wavelength. We inserted the values of RI and length compaction in to our FBG model for a fast neutron fluence of $2.4\times 10^{21}\;n/{\mathrm{cm}}^{2}$ at a temperature of 95 °C. First, we simulated the FBG without any radiation exposure. The spectrum of FBG before irradiation is shown in Figure 6 (black line), and it can be seen that the Bragg wavelength is at 1549.9 nm. Then, we considered the radiation effect on the Bragg wavelength by inserting only RI change obtained by Equation (6) due to a fast neutron fluence of $2.4\times 10^{21}\;n/{\mathrm{cm}}^{2}$ at a temperature of 95 °C [9]. Please note that we did not consider the RIA induced RI change as it’s quite complex and requires the full spectrum of light. It can be observed that Bragg wavelength gives a redshift of 10.62 nm (blue line) due to the increased RI induced by radiation. In a similar way, we input the radiation-induced grating period (linear compaction) change into our model while considered no change in RI. It is seen that compacted length provides a blueshift of 11.31 nm (green line). It is well known that increased RI shifts the Bragg wavelength to the longer wavelength side, whereas a compacted grating period shifts it to the shorter wavelength side according to Equation (11). Then, we inserted the radiation-induced change both in the RI and the grating period in our FBG model and ran a simulation. An overall shift of 0.77 nm (red line) to the shorter wavelength side can be observed, which indicates the effect of length compaction dominates over the effect of RI. It is interesting to see that there is no reduction of Bragg amplitude due to the radiation which is not the normal case. Experimental results indicate that radiation reduced the amplitude of the Bragg peak [9,21], and the possible reasons might the defect generation and eraser of gratings. As we did not consider any RIA in our simulation, the amplitude of the Bragg peak slightly increased due to the increased RI. An overall shift of 0.77 nm led to a temperature error of 77 °C by assuming the FBG temperature sensitivity coefficient of 10 pm/°C. It is also possible that RIA induced change in RI may further accelerate the errors. By knowing the macroscopic changes that cause a shift of 0.77 nm for FBG, it is possible to correct the drift.

The alteration of RI may occur due to RIC, RIA, temperature, and so on in a radiation environment. However, Lorentz–Lorenz or point dipole theory only helps to calculate compaction (or density) induced RI change. To understand the whole picture, it is crucial to know the RI change caused by all possible reasons (RIA, RIC, irradiation temperature, dopant diffusion, etc.) due to radiation. An in-line measurement of macroscopic changes, no matter what it causes, provides the actual change in RI and length, which can be then used to correct the sensor drift.

## 5. Thermal Effects on Cavities

In this section, the thermal effects on cavities, as well as gas RI is considered, if the air cavity is filled with helium gas. From now on, the air cavity will be called a gas cavity for better understanding, since it is filled with helium gas. As the thermo-optic and the thermal expansion coefficients are well defined for a pure silica-based cavity, we mainly investigate the thermal effects on the gas cavity. Temperature relates to the pressure through the ideal gas law expressed by

(14) PV=mRT

where *P*, *V*, *m*, *R*, and *T* are the pressure, volume, amount of gas, ideal gas constant, and temperature, respectively. If the diameter of the gas cavity is 40 µm (inner diameter of silica capillary tube) and as the cavity length of the gas cavity (117 µm) is known, *V* and hence, *m* can be calculated at normal temperature and pressure (NTP). Then, pressure *P*_2_ at temperature *T*_2_ (300 °C in our case for example) is calculated. Once pressure is known, strain and hence the change in length can be determined by using

(15) $$\Delta L=\varepsilon \times L=\frac{P}{E}\times L=\frac{FL}{AE}$$

where ε, E, F, and A are the, strain, Young’s modulus, applied force to the gas cavity, and cross-sectional area of glass, respectively. Since the overall radius of the capillary tube is 62.5 µm and the radius of the glass in the capillary tube is 20 µm, then $A=\pi \left(62.5^{2}-20^{2}\right)\times 10^{-12}\;m^{2}$. The force acting on the glass due to pressure is $F≅P\times \pi \times 20^{2}\times 10^{-12}\;m^{2}$. As the Young’s modulus of the bare silica fiber is 69.22 ± 0.42 GPa [55], strain-induced length change ΔL can be calculated by performing some iterations until the result becomes convergent. We obtained strain induced ΔL of 0.038 nm at 300 °C, which is very small compared to the thermally induced ΔL of 11.43 nm of pure silica fiber, considering the thermal expansion coefficient of $4.1\times 10^{-7}/{}^{\circ}C$. As a result, pressure induced length change can be neglected compared to the thermally induced one.

Next, we emphasize the temperature effect on the RI of helium gas. The RI of gas can be determined from the Lorentz–Lorenz equation, and it can be written in the form of

(16) $$\frac{n^{2}-1}{n^{2}+2}=A_{R}\rho_{m}+B_{R}\rho_{m}{}^{2}$$

where $\rho_{m}$ is the density in moles per unit volume, $A_{R}$ is the molar polarizability, and $B_{R}$ is the second refractivity virial coefficient. To calculate the density $\rho_{m}$, the *m* is calculated first at NTP since *V* is known. The molar refractivity $A_{R}$ for helium gas is calculated using the following expression in [56]

(17) $$A_{R}=0.51725407+\frac{1197.5410}{\lambda^{2}}+\frac{3.290677\times 10^{6}}{\lambda^{4}}+\frac{9.800874\times 10^{9}}{\lambda^{6}}$$

where λ is the wavelength of light used. The refractivity virial coefficient $B_{R}$ is calculated using the expression suggested by [56]:

(18) $$B_{R}=-0.032-0.0001T$$

Please note that the above expression for $B_{R}$ was developed at a wavelength of 633 nm [56]. However, we used this value of $B_{R}$ at 1550 nm, since $B_{R}$ has only a small effect on the refractive index (modify the result less than $2\times 10^{-10}$) [56]. Then, the value of each parameter is inserted into Equation (16), and the refractive index *n* is calculated. For high temperature *T*_2_ (300 °C in our case), thermally induced ΔL and hence the new length is calculated using the thermal expansion coefficient of pure silica. New volume is then determined from the new length assuming other parameters remain unchanged. Based on the new volume, density $\rho_{m}$ and RI *n* are calculated at 300 °C. The obtained difference in RIs between NTP and 300 °C is $7.4\times 10^{-9}$, which is very small and can be neglected. We investigated the strain induced length change and temperature induced RI of the gas cavity and found that both are very small and can be neglected. The only parameter remains that dominates on the interferometric fringe of the gas cavity is the thermally induced length expansion.

We believe the active compensation technique can also be used to calculate the thermo-optic and thermal expansion coefficients of silica-based fibers in a temperature environment. In a similar way discussed for radiation effect, the thermal expansion from the air cavity can be used to calculate the change in RI in the silica cavity.

## 6. Conclusions

A simple cascaded FPI has been proposed to calculate the RI and length compaction. The air cavity is used to calculate the linear compaction, and then the RI is calculated from the silica cavity by considering the same amount of linear compaction of this cavity to that of the air cavity. However, cavity length separation for each cavity is required to perform this job. The FFT algorithm has been utilized to get the frequency domain signal from the total interference spectrum of the cascaded FPI. Then, two bandpass filters have been applied to separate the individual cavity lengths from the total spectrum. This simple active compensation technique by using just two cascaded cavities can be used to measure RI due to RIC, RIA, temperature, pressure, dopant diffusion, or any combination of them.

## Figures and Tables

**Table 1:** Original and retrieved lengths of cavities of the cascaded FPI.

Cavity	Original Cavity Length (µm)	Retrieved Cavity Length (µm)
Air cavity	117	117.39
Silica cavity	211	210.69

**Table 2:** Reconstructed cavity lengths before and after irradiation to a fast fluence of $2.4\times 10^{21}\;n/{\mathrm{cm}}^{2}$ at a temperature of 95 °C.

Cavity	Before Irradiation Retrieved Original Cavity Length (µm)	After Irradiation Retrieved Compacted Cavity Length (µm)
Air cavity	117.39	116.56
Silica cavity	210.69	209.20

## Data Availability

Not applicable.
